# Scaling Peak Oxygen Consumption for Body Size and Composition in People With a Fontan Circulation

**DOI:** 10.1161/JAHA.122.026181

**Published:** 2022-12-14

**Authors:** Curtis A. Wadey, Alan R. Barker, Graham Stuart, Derek L. Tran, Karina Laohachai, Julian Ayer, Rachael Cordina, Craig A. Williams

**Affiliations:** ^1^ Children’s Health & Exercise Research Centre (CHERC) Public Health and Sport Science, Faculty of Health and Life Sciences, University of Exeter Exeter United Kingdom; ^2^ Bristol Congenital Heart Centre, The Bristol Heart Institute, University Hospitals Bristol NHS Foundation Trust Bristol United Kingdom; ^3^ Central Clinical School, The University of Sydney Camperdown New South Wales; ^4^ Department of Cardiology Royal Prince Alfred Hospital Camperdown New South Wales; ^5^ Heart Research Institute, Charles Perkins Centre, The University of Sydney Camperdown New South Wales

**Keywords:** allometry, cardiorespiratory fitness, congenital heart disease, CPET, Basic Science Research, Exercise, Congenital Heart Disease

## Abstract

**Background:**

Peak oxygen consumption (peak $\dot{V}O_{2}$) is traditionally divided (“ratio‐scaled”) by body mass (BM) for clinical interpretation. Yet, it is unknown whether ratio‐scaling to BM can produce a valid size‐independent expression of peak $\dot{V}O_{2}$ in people with a Fontan circulation. Furthermore, people with a Fontan circulation have deficits in lean mass, and it is unexplored whether using different measures of body composition may improve scaling validity. The objective was to assess the validity of different scaling denominators (BM, stature, body surface area, fat‐free mass, lean mass, and appendicular lean mass using ratio and allometric scaling).

**Methods and Results:**

Eighty‐nine participants (age: 23.3±6.7 years; 53% female) with a Fontan circulation had their cardiorespiratory fitness and body composition measured by cardiopulmonary exercise testing and dual‐energy x‐ray absorptiometry. Ratio and allometric (log‐linear regression) scaling was performed and Pearson correlations assessed scaling validity. Scaling denominators BM (*r*=−0.25, *P*=0.02), stature (*r*=0.46, *P*<0.001), and body surface area (0.23, *P*=0.03) were significantly correlated with their respective ratio‐scaled expressions of peak $\dot{V}O_{2}$, but fat‐free mass, lean mass, or appendicular lean mass were not (*r*≤0.11; *R* ^2^=1%). Allometrically expressed peak $\dot{V}O_{2}$ resulted in no significant correlation with any scaling denominator (*r*=≤0.23; *R* ^2^=≤4%).

**Conclusions:**

The traditional and accepted method of ratio‐scaling to BM is invalid because it fails to create a size‐independent expression of peak $\dot{V}O_{2}$ in people with a Fontan circulation. However, ratio‐scaling to measures of body composition (fat‐free mass, lean mass, and appendicular lean mass) and allometric techniques can produce size‐independent expressions of peak $\dot{V}O_{2}$ in people with a Fontan circulation.

**Nonstandard Abbreviations and Acronyms**
ALM: appendicular lean mass
BIA: bioimpedance analysis
BM: body mass
ConHD: congenital heart disease
CPET: cardiopulmonary exercise test
ESC: European Society of Cardiology
FFM: fat‐free mass
LM: lean mass
Peak $\dot{V}O_{2}$
: peak oxygen consumption

**Clinical Perspective**

**What Is New?**

- Ratio‐scaling peak oxygen consumption to body mass (mL·kg^−1^·min^−1^) is spurious, because it fails to create a size‐independent expression of fitness in people with a Fontan circulation, thus questioning its validity.
- Ratio‐scaling peak oxygen consumption to body mass results in a statistical artifact where Fontan patients with lower body mass have an artificially inflated fitness score, and Fontan patients who are heavier have a suppressed fitness score.
- Dividing peak oxygen consumption by measures of body composition such as fat‐free mass or allometric scaling can provide valid size‐independent expressions of fitness.

**What Are the Clinical Implications?**

- Caution is advised when interpreting ratio‐scaled peak oxygen consumption in people with a Fontan circulation, especially if considering medical intervention.
- Fat‐free mass should also be considered as an alternative scaling denominator.

The Fontan circulation is created by a series of palliative operations used in children with univentricular congenital heart disease (ConHD), such as tricuspid atresia or hypoplastic left heart syndrome.^1^ The procedure connects the systemic veins to the pulmonary arteries, to reduce cardiac volume loading and increase oxygen saturation.^1^

Patient life expectancy has improved substantially since the first operations were performed in 1968, and currently >90% of children with a Fontan circulation will survive until adulthood.^2^ Recent epidemiological models derived from data collected in 8 European nations, the United States, Australia, and New Zealand have reported that there are ≈48 000 people living with a Fontan circulation, which is expected to rise to 59 000 by 2030.^3^ However, the Fontan procedure is not a panacea, because this unique circulation creates a bottleneck within the pulmonary vasculature,^4^ which over time results in sequelae such as arrhythmia, protein‐losing enteropathy, reduced exercise capacity, heart failure, and reduced life expectancy.^2^, ^4^, ^5^

The European Society of Cardiology and the American Heart Association guidelines recommend that exercise capacity should be assessed routinely (European Society of Cardiology annual test; American Heart Association every 1–2 years) as a part of long‐term follow‐up in people with a Fontan circulation.^6^, ^7^ Peak oxygen consumption (peak $\dot{V}O_{2}$) is an objective measure of exercise capacity,^8^ and lower levels of peak $\dot{V}O_{2}$ have been directly associated with major adverse cardiovascular events and health‐related quality of life in people with a Fontan circulation.^9^, ^10^

Peak $\dot{V}O_{2}$ is determined by the Fick equation (peak $\dot{V}O_{2}$=cardiac output * arteriovenous oxygen content difference).^11^ Traditionally, clinicians and researchers have indexed or “ratio‐scaled” absolute peak $\dot{V}O_{2}$ (mL·min^−1^) to body mass (BM) (mL·kg^−1^·min^−1^), to create a size‐independent expression of peak $\dot{V}O_{2}$ to assess intra‐ and interindividual exercise capacity. In clinical settings, a ratio‐scaled expression of peak $\dot{V}O_{2}$ (mL·kg^−1^·min^−1^) is then converted into percent of predicted value to risk stratify patients or used as a predictor variable in prognosis or outcome‐based research.^12^, ^13^, ^14^, ^15^

Ratio‐scaling relies on a statistical assumption that the relationship between peak $\dot{V}O_{2}$ and BM is directly proportional.^16^ This assumption is not commonly met in exercise physiology, which produces a statistical artifact that results in an artificially inflated peak $\dot{V}O_{2}$ in lighter individuals and an artificially deflated peak $\dot{V}O_{2}$ in heavier individuals.^17^, ^18^ Therefore, purely because of a statistical artifact, some individuals' exercise capacity artificially appears better and, for others, it is worse.^16^, ^19^ Invalid ratio‐scaled measures of peak $\dot{V}O_{2}$ have produced spurious associations to health outcomes (because of the residual effect of body size), and once peak $\dot{V}O_{2}$ has been scaled appropriately these associations have been weakened or removed entirely.^20^ Often in clinical research there is no statistical justification provided for ratio‐scaling peak $\dot{V}O_{2}$ to BM.^16^, ^18^ Furthermore, the standard practice of ratio‐scaling to BM does not account for the individual variations in body composition.

Fat‐free mass (FFM) and lean mass (LM) have a stronger association with absolute peak $\dot{V}O_{2}$ when compared with BM, and it is currently recommended practice to scale peak $\dot{V}O_{2}$ to LM.^21^, ^22^, ^23^, ^24^, ^25^ Tran et al^26^ and Powell et al^27^ have recently characterized body composition using dual‐energy x‐ray absorptiometry and bioimpedance analysis in people with a Fontan circulation. They describe an unfavorable body composition profile with lower LM (“Fontan‐associated myopenia”), which was associated with lower levels of muscular strength, cardiac function, and absolute peak $\dot{V}O_{2}$ (mL·min^−1^).

Considering the unique “Fontan‐associated” myopenic body composition,^26^, ^27^ ratio‐scaling peak $\dot{V}O_{2}$ to BM (mL·kg^−1^·min^−1^), may be shrouding important physiological relationships and health‐related outcomes in the current literature. These limitations can be overcome with the use of different scaling denominators (ie, LM) and using mathematical techniques such as allometric scaling.^28^ Allometric scaling produces a size‐independent measure of peak $\dot{V}O_{2}$ by using a power function to describe the relationship between peak $\dot{V}O_{2}$ and the scaling denominator.^17^ This technique has been established in other areas such as heart failure and cystic fibrosis research^29^, ^30^ but has yet to be applied to ConHD. A valid size‐independent expression of peak $\dot{V}O_{2}$ is essential to understanding the associations between exercise capacity and health outcomes and the effectiveness of health interventions.

The purpose of this study was to examine how peak $\dot{V}O_{2}$ can be adjusted for using different body size and composition variables in people with a Fontan circulation, with a view to providing guidance for the reporting of peak $\dot{V}O_{2}$ in future research and clinical practice. Specifically, the aims of this study were (1) to assess the validity of the currently accepted ratio‐scaling of peak $\dot{V}O_{2}$ to body mass and (2) to assess the validity of ratio and allometric scaling using a range of body size variables in people with a Fontan circulation.

## METHODS

### Participants and Study Design

The current study performed secondary data analysis on anonymized individual participant data made available by Tran and colleagues and the ANZ (Australian and New Zealand Fontan Registry).^26^, ^31^, ^32^ Ethics approval was granted for the data collection by the relevant Ethics Review Committee at each participating center, informed consent was obtained from all participants, and data transfer agreements were approved by the ANZ Fontan Registry Steering Committee in 2021. The data that support the findings of this study are available from R. Cordina upon reasonable request.

### Experimental Measures and Protocol

All methods relating to the collection of participant data have been previously described in the original publications.^26^, ^31^, ^32^ In summary, peak $\dot{V}O_{2}$ was measured by a cardiopulmonary exercise test, using an individualized ramp protocol on an electronically braked cycle ergometer, and body composition was assessed via dual‐energy x‐ray absorptiometry.

Body size variables considered as a potential scaling denominator were BM, stature, body surface area (BSA), FFM, LM, and appendicular lean mass (ALM). BSA was calculated using the DuBois equation and measured in meters squared (m^2^),^33^ Stature was reported in centimeters (cm), and all other variables (BM, FFM, LM, and ALM) were reported in kilograms (kg). FFM was calculated by summing the soft tissue LM and total bone mineral content, and ALM was calculated by summing the LM of the arms and legs.

### Scaling Approaches

Absolute peak $\dot{V}O_{2}$ was expressed relative to each body size parameter via ratio‐scaling (Y/X, ie, mL·BM^−1^·min^−1^). Allometric scaling was performed by transforming the scaling numerator (ie, peak $\dot{V}O_{2}$ mL·min^−1^) and denominator (body size variable) to its natural logarithm (ln), followed by linear regression analysis with sex included as a covariate $\begin{array}{c} \left(\ln\;\text{Peak}\;\dot{V}O_{2}=\beta_{0}+\beta_{1}\ln\;\text{body size variable}+\beta_{2}\mathrm{sex}\right) \end{array}$. This provided an estimate of the relationship (β estimates [b]) between peak $\dot{V}O_{2}$ and each body size variable and the confidence around this estimate (95% CI). The assumptions for linear regression analysis (continuous data, no interaction between the independent variables, normal distribution, independence, linearity, and homoscedasticity of the residuals) were checked using scatter plots, histograms, and the Durbin‐Watson statistic. Allometric expressions of peak $\dot{V}O_{2}$ were produced by dividing absolute peak $\dot{V}O_{2}$ (mL·min^−1^) by each scaling denominator, with the addition of the respective power function (b value) identified from the log‐linear regression (Y/X^b^, ie, mL·BM^b^·min^−1^).

### Statistical Analysis

Descriptive continuous data are represented as means± SD. Descriptive categorical data are provided as count data and percentages. The allometric scaling b exponents and 95% CIs have been reported and Pearson correlation coefficients assessed model validity for both males and females separately. Scaled or indexed peak $\dot{V}O_{2}$ (eg, mL·FFM^−1^·min^−1^) should have a negligible and nonsignificant correlation with the respective body size variable (eg, FFM), to be considered a valid scaling approach. Pearson correlation coefficients (r) were interpreted as <0.2, negligible, 0.2 to 0.49, small, 0.5 to 0.79, moderate, and ≥0.8, large.^34^

To assess how ratio and allometric scaling affects individual participants’ peak $\dot{V}O_{2}$ relative to the whole cohort, the participants were ordered from highest to lowest peak $\dot{V}O_{2}$ and were assigned a rank position (most fit=1 to least fit=89). Participant rank was then compared between ratio and allometrically scaled expressions of peak $\dot{V}O_{2}$ using the “Vlookup” function in Microsoft Excel (Version 2101, Microsoft Corporation, Redmond, WA). This reported the number of participants whose rank changed because of allometric scaling and the magnitude of the change.

The α level was set at 0.05, all statistical analyses were conducted using IBM SPSS statistics (Version 28.0; IBM, Armonk, NY), and figures were produced using GraphPad (Version 9; GraphPad Software, Inc., San Diego, CA). An example of statistical code used for the allometric scaling can be found in the supplemental material, and the participants’ data are available from the author upon reasonable request.

## RESULTS

### Participants

**Figure 1:**
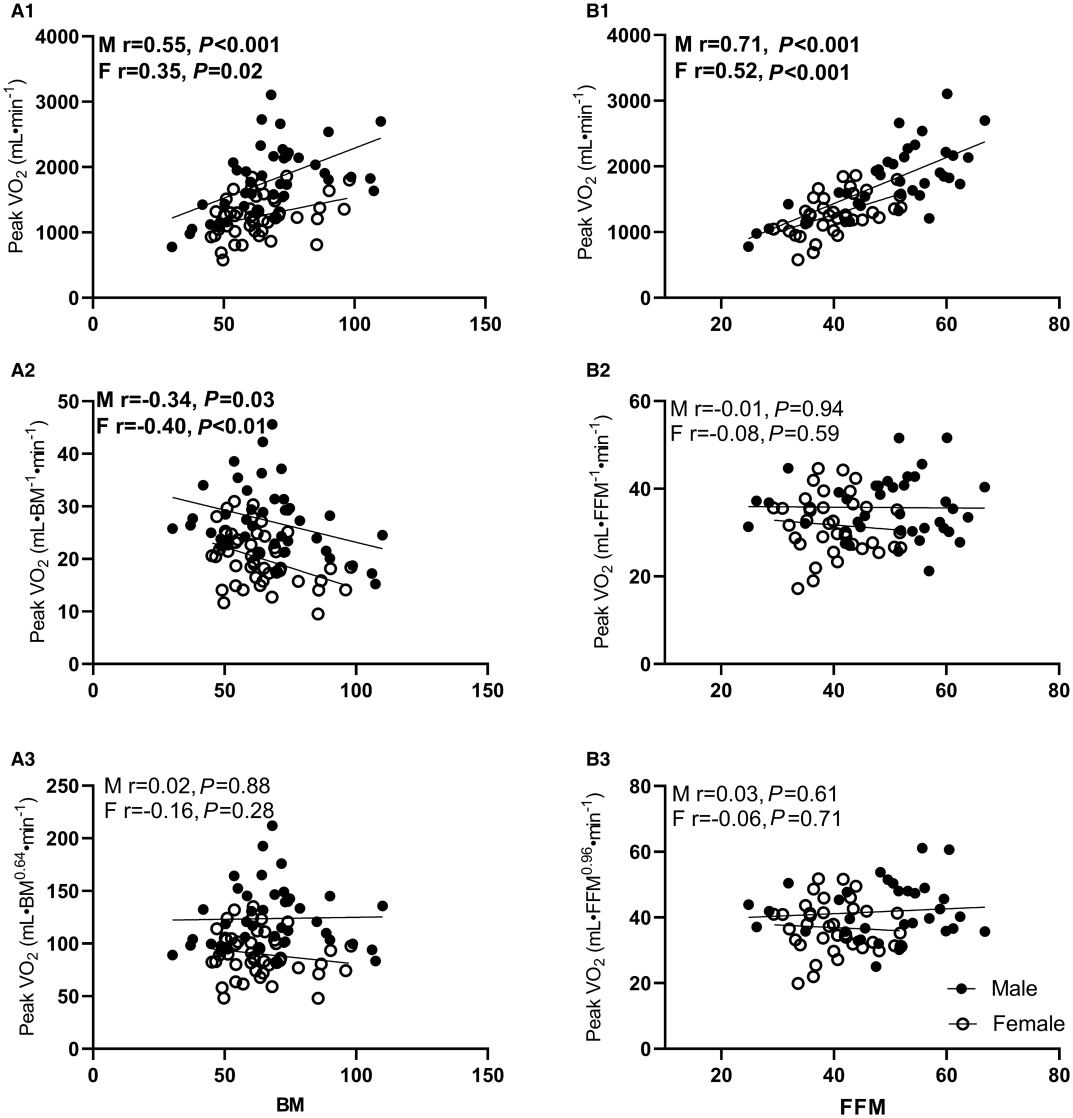
Scaling peak VO_2_ to BM and FFM using ratio and allometric scaling. **A1** & **B1** indicates absolute peak oxygen consumption; **A2**, ratio‐scaled peak oxygen consumption to BM; **B2**, ratio‐scaled peak oxygen consumption to FFM; **A3**, allometrically scaled peak oxygen consumption to BM; **B3**, allometrically scaled peak oxygen consumption to FFM. BM indicates body mass; F, female; FFM, fat‐free mass; M, male; peak VO_2_ (mL·min^−1^), peak VO_2_ (mL·BM^−1^·min^−1^), peak VO_2_ (mL·FFM^−1^·min^−1^), peak VO_2_ (mL·BM^0.64^·min^−1^), and peak VO_2_ (mL·FFM^0.96^·min^−1^).

Participant characteristics are reported in Table 1. Peak $\dot{V}O_{2}$ was ratio and allometrically scaled to the different body size parameters, the scaling exponents used are reported in Table 2, and the scaling validity is reported in Figure 1, Figure S1, and Table S1.

**Table 1:** Participant Characteristics

Patient characteristics	All participants (n=89)	Males (n=42)	Females (n=47)
	Mean±SD, n (%)		
Demographics			
Age, y	23.3±6.7	21.6±6.1	26.4±7.7
Dominant ventricle			
Left ventricle	54 (61)	25 (60)	29 (62)
Indeterminate	4 (4)	3 (7)	1 (2)
Biventricular	7 (8)	4 (9)	3 (6)
Right ventricle	24 (27)	10 (24)	14 (30)
Type of Fontan			
APC	12 (13)	2 (5)	10 (21)
LT	23 (26)	9 (21)	14 (30)
ECC	54 (61)	31 (74)	23 (49)
Body composition			
Body mass, kg	65.2±16.7	67.6±18.8	63.5±13.1
Stature, cm	165.2±10.3	170.3±10.2	161.1±7.9
BMI, kg/m^2^	23.7±5.0	22.7±0.8	24.5±0.7
BSA,m^2^	1.7±0.2	1.8±0.3	1.6±0.2
FFM, kg, (n=77, M=38, F=39)	44.9±9.6	49.7±10.4	40.2±5.7
Total LM, kg	42.7±9.2	47.8±9.9	38.9±5.8
ALM, kg	17.8±4.5	20.4±4.6	15.6±2.6
CPET performance			
Resting SpO_2_ (%) (n=82)	93±4	93±3	93±4
Peak SpO_2_ (%) (n=81)	90±5	90±5	90±5
Peak HR	152±26	160±25	146±26
Peak HRR (n=81)	67±27	71±26	65±27
Peak $\dot{V}O_{2}$ (mL·min^−1^)	1506±502	1792±527	1252±305
Peak $\dot{V}O_{2}$ (mL·kg·^−1^min^−1^)	23.5±6.9	27.1±6.7	20.1±5.1
$\dot{V}O_{2}$ at the GET (mL·min^−1^) (n=84)	1034±398	1200±458	885±261
Peak RER	1.16±0.10	1.15±0.09	1.18±0.11
Peak work rate (W)	120±39	139±44	104±23

ALM indicates appendicular lean mass; APC, aortopulmonary collateral; BMI, body mass index; BSA, body surface area; CPET, cardiopulmonary exercise test; ECC, extracardiac conduit; FFM, fat‐free mass; GET, gas exchange threshold; HR, heart rate; HRR, heart rate reserve; LM, lean mass; LT, lateral tunnel; RER, respiratory exchange ratio; SpO_2_, oxygen saturation; and $\dot{V}O_{2}$, volume of oxygen.

**Table 2:** Scaling Exponents

Body composition	b estimate	95% CI	*R*
BM, kg	0.64	0.43–0.85	0.49
Stature, cm	2.58	1.62–3.54	0.45
BSA, m^2^	1.26	0.88–1.63	0.51
FFM, kg, (n=77)	0.96	0.72–1.20	0.60
LM, kg	0.88	0.63–1.12	0.54
ALM, kg	0.89	0.68–1.10	0.61

ALM indicates appendicular lean mass; BM, body mass; BSA, body surface area; FFM, fat‐free mass; and LM, lean mass.

### Scaling

**Figure 2:**
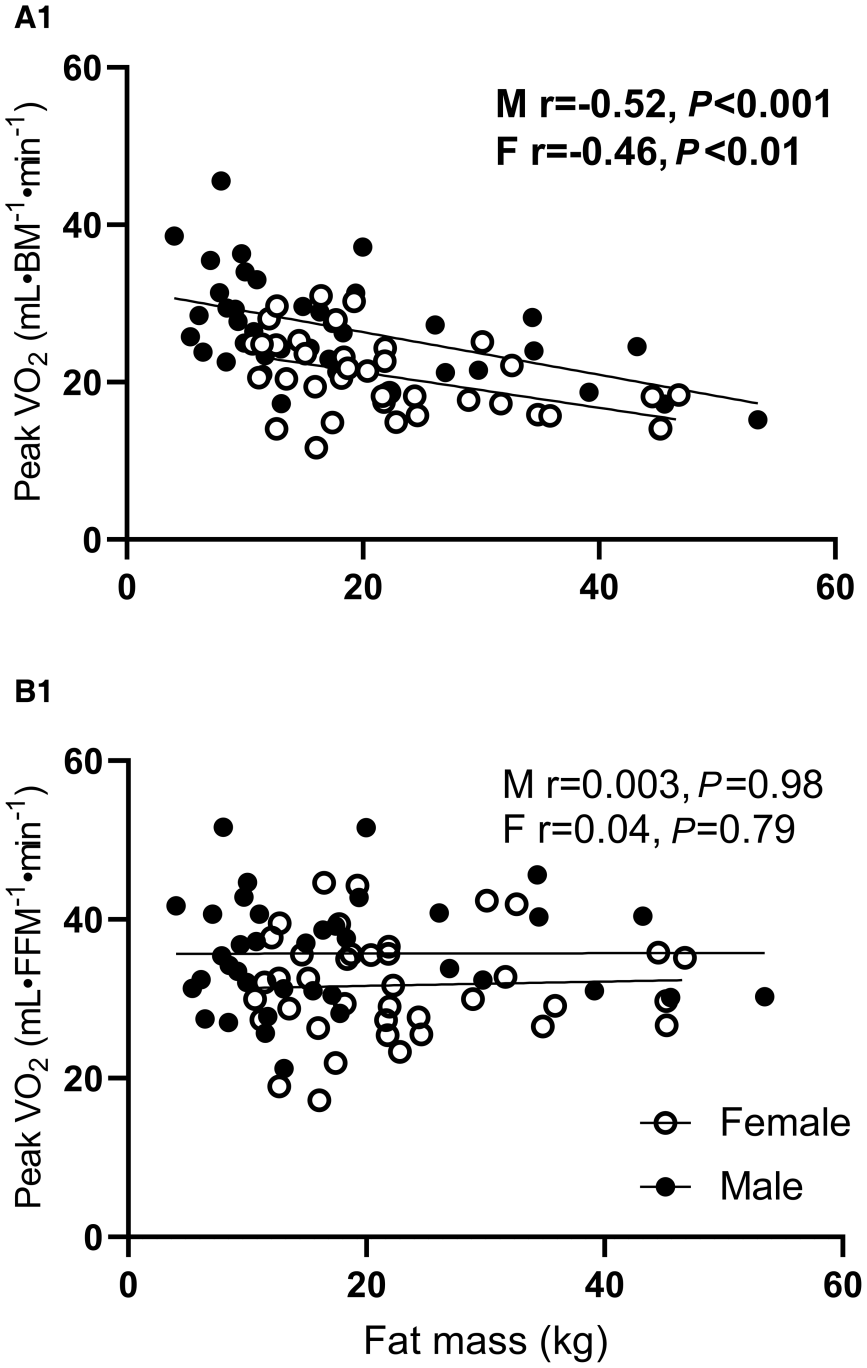
Relationship between ratio‐scaled peak VO_2_ and fat mass. **A1**, Peak VO_2_ (mL·BM^−1^·min^−1^), ratio‐scaled peak oxygen consumption to body mass; **B1**, Peak VO_2_ (mL·FFM^−1^·min^−1^), ratio‐scaled peak oxygen consumption to fat‐free mass; F, female; and M, male.

Participants had a mean absolute peak $\dot{V}O_{2}$ of 1506±502 mL·min^−1^. Absolute peak $\dot{V}O_{2}$ (mL·min^−1^) had a moderate to strong positive correlation with all body size variables (BM, *r*=0.47 [95% CI, 0.29–0.62]; stature, *r*=0.59 [95% CI, 0.44–0.71]; BSA (*r*=0.57 [95% CI, 0.42–0.69]); FFM (*r*=0.75 [95% CI, 0.63–0.83]); LM (*r*=0.71 [95% CI, 0.58–0.79]); ALM (*r*=0.76 [95% CI, 0.66–0.84]; *P*<0.001). Body size variables BM (*r*=−0.25 [95% CI, −0.43 to −0.04], *P*=0.02), stature (*r*=0.46 [95% CI, 0.28–0.61], *P*<0.001), and BSA (*r*=0.23 [95% CI, 0.20–0.42], *P*=0.03) were significantly correlated with their respective ratio‐scaled expressions of peak $\dot{V}O_{2}$, but body size variables FFM (*r*=0.11 [95% CI, −0.12 to 0.33], *P*=0.33), LM (*r*=0.08 [95% CI, −0.13 to 0.29]), *P*=0.44), or ALM (*r*=0.03 [95% CI, −0.18 to 0.23], *P*=0.81) were not. Allometrically expressed peak $\dot{V}O_{2}$ resulted in negligible nonsignificant correlations with any body size variable for the whole group (BM, *r*=0.04 [95% CI, −0.17 to 0.24]); stature (*r*=0.17 [95% CI, −0.04 to 0.37]; BSA (*r*=0.11 [95% CI, −0.10 to 0.31]); FFM, *r*=0.15 [95% CI, −0.07 to 0.36]); LM (*r*=0.20 [95% CI, −0.06 to 0.39]); and (ALM, *r*=0.16 [95% CI, −0.05 to 0.36]). The relationships and scaling validity between all expressions of peak $\dot{V}O_{2}$ and all body size variables are reported in Table S1 and Figure S1 for both males and females. Ratio‐scaled peak $\dot{V}O_{2}$ to BM has a significant negative correlation with both BM and fat mass (Figures 1 and 2). However, peak $\dot{V}O_{2}$ scaled to FFM has no correlation with BM or fat mass (Figure 2).

**Figure 3:**
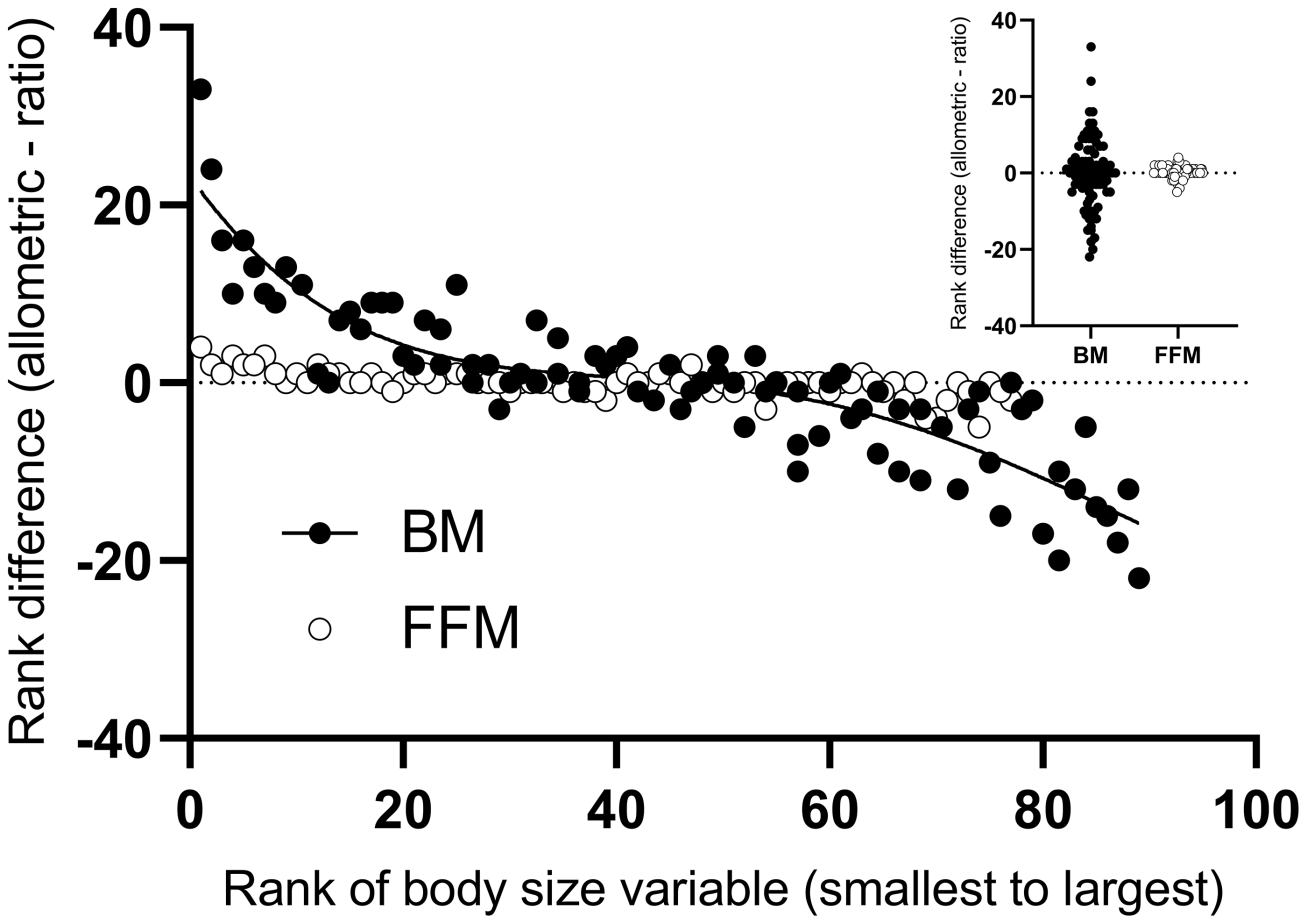
Rank difference between allometric and ratio scaling as a function of rank body size. BM indicates body mass; and FFM, fat‐free mass.

Table 3 reports the number of participants whose individual rank changed and the average number of places they moved once peak $\dot{V}O_{2}$ was allometrically scaled. Fewer participants maintained their fitness rank once allometrically scaled when the scaling denominators were BM (11%) or stature (7%) compared with BSA, FFM, LM, and ALM (21%–45%). Furthermore, scaling for BSA, FFM, LM, and ALM resulted in fewer rank changes (2–4 versus 7–8 ranks). Figure 3 presents the individual participant data of the rank difference between allometric and ratio‐scaling, as a function of rank body size. Positive numbers indicate that ratio‐scaling inflated the fitness rank of an individual and negative numbers indicate that ratio‐scaling penalized individuals. Values close to zero indicate little‐to‐no change in ratio‐scaling rank compared with the allometric model. Those with a body size rank further away from the average rank were the most susceptible to rank change. Lighter individuals had an inflated value, and heavier individuals had a suppressed value when BM was the scaling denominator. For example, the smallest individual was classified 33 ranks (out of 89 ranks) higher, and the heaviest individual was ranked 22 places lower in the ratio‐scaled compared with the allometrically scaled expression of fitness (rank difference range −22 to 33). Using FFM as a scaling denominator improved the misclassification of fitness rank (rank difference range −5 to 4) between the scaling approaches.

**Table 3:** Participant Rank Changes From Ratio Scaling to Allometric Scaling Within a Body Size Variable

	BM	Stature	BSA	FFM (n=77)	LM	ALM
N rank maintained	10 (11%)	6 (7%)	22 (25%)	35 (45%)	22 (25%)	19 (21%)
N rank changed	79 (89%)	83 (93%)	67 (75%)	42 (55%)	67 (75%)	70 (79%)
Avg. n of rank positions changed	7	8	4	2	3	3
N of participants (%)						

ALM indicates appendicular LM; Avg., average; BM, body mass; BSA, body surface area; FFM, fat‐free mass; and LM, lean mass.

## DISCUSSION

This is the first study to assess the scaling of peak $\dot{V}O_{2}$ to different body size and composition variables in people with ConHD. Important findings from the current study are the following: (1) the currently accepted ratio‐scaling approach of normalizing peak $\dot{V}O_{2}$ to BM is invalid because it is still biased by body size, (2) allometric scaling was able to produce valid size‐independent expressions of peak $\dot{V}O_{2}$ irrespective of scaling denominator, (3) ratio‐scaling peak $\dot{V}O_{2}$ to body size variables that reflect body composition (eg, FFM, LM, and ALM) were valid scaling approaches and were not biased by body size and composition.

Ratio‐scaling peak $\dot{V}O_{2}$ to BM is an invalid technique, because it fails to create a size‐free expression of peak $\dot{V}O_{2}$.^17^ This may have several potential consequences: First, heavier individuals whose peak $\dot{V}O_{2}$ is artificially suppressed could potentially undergo unnecessary investigations/interventions (vice‐versa for lighter individuals). Second, individuals who participate in interventions and who lose fat mass will have an increase in their peak $\dot{V}O_{2}$ independent of any cardiorespiratory adaptations. Third, the lack of a size‐independent expression of peak $\dot{V}O_{2}$ may be altering associations between cardiorespiratory fitness and health outcomes.^10^, ^35^

In contrast to the currently accepted but invalid approach of ratio‐scaling to BM, all expressions of allometrically scaled peak $\dot{V}O_{2}$ were not correlated with their respective scaling denominator. Therefore, allometry can produce valid size‐independent expressions of peak $\dot{V}O_{2}$, which can then be applied in a variety of clinical and research settings. However, ratio‐scaling denominators more reflective of body composition (FFM, LM, and ALM) also produced negligible nonsignificant correlations. This suggests that ratio‐scaling to specific measures of body composition can be effective in creating valid size‐independent expressions of peak $\dot{V}O_{2}$, which can be easily applied within a clinical setting. Body composition variables such as FFM were able to produce a valid ratio‐scaled expression peak $\dot{V}O_{2}$ because the b value identified in the allometric model was 0.96, which is close to the ratio‐scaling assumption of direct proportionality (ie, b value of 1). A potential recommendation to clinical exercise physiologists could be to additionally report peak $\dot{V}O_{2}$ ratio‐scaled to FFM, because this removes potential influences from changes in fat mass during long‐term follow‐up.

People with a Fontan circulation have a decreased lean BM and an increased percentage body fat (≈15%) compared with healthy controls. This supports the proposition that body composition should be accounted for when assessing this unique population.^27^ In the current study, the FFM of the participants was measured by dual‐energy x‐ray absorptiometry.^26^ Bioimpedance analysis is an alternative method for assessing body composition, which is less expensive, quicker, and requires less training and space compared with dual‐energy x‐ray absorptiometry.^36^, ^37^ Bioimpedance analysis has been previously used in Fontan populations^27^ and has been reported as safe in individuals who have cardiac devices in situ.^38^, ^39^ However, any method that indirectly estimates body composition such as bioimpedance analysis or skinfold may need to be validated in this population because of their unique body composition.^26^, ^40^

Understanding the assumptions of ratio‐scaling physiological data will improve the planning and evaluation of clinical trials. The current standard of ratio‐scaling to BM is biased on body size and composition; therefore, studies that have longer durations where patient body size and composition change during the follow‐up (eg, growing pediatric cohorts) could be aided by scaling to measures of body composition and/or allometric scaling. Furthermore, valid statistical scaling methods are vital in the diagnosis and risk stratification of individuals. Peak $\dot{V}O_{2}$ (ratio‐standard to BM) has been reported as being able to identify young people at risk of cardiometabolic disease; however, once body size and composition were controlled for this association diminished.^20^ This suggests that individuals would have been identified at an increased risk who were not at risk (false positive) and that body composition is shrouding the associations reported. Ejection fraction (ratio‐scaled metric) has also been reported as biasing patient diagnoses by misclassifying healthy individuals with heart failure who have large end‐diastolic volumes.^30^ Therefore, ratio‐scaling to measures of body composition and/or allometric techniques improves the clinical utility of peak $\dot{V}O_{2}$ as an outcome in clinical research because they are size and composition independent.

A limitation of this study is that allometric scaling cannot practically be introduced into a clinical setting without considerable reorganizing of data reporting. However, allometric scaling can produce size‐independent expressions of peak $\dot{V}O_{2}$ and should be considered when analyzing physiological data in clinical research. Allometric scaling has been successfully utilized in other clinical populations who have an altered body composition, such as people with cystic fibrosis^29^, ^41^ but not yet in people with other types of ConHD. The scaling coefficients derived from the current study should not be applied to other populations. Instead, researchers should derive their own allometric coefficients.^42^

## CONCLUSIONS

The traditional and accepted method of ratio‐scaling peak $\dot{V}O_{2}$ to BM is invalid. Ratio‐scaling measures of body composition and allometrically scaling to any body size variable produces valid size‐independent expressions of peak $\dot{V}O_{2}$ in people with a Fontan circulation. It is unknown whether allometric methods are effective in other subtypes of ConHD and whether the prognostic and predictive abilities of peak $\dot{V}O_{2}$ are maintained once it is appropriately scaled. Although further studies are required to replicate and extend these findings, they suggest that peak $\dot{V}O_{2}$ should be scaled to estimates of FFM within clinical practice and research.

## Sources of Funding

C. Wadey is funded by an industrial PhD scholarship by Canon Medical Systems UK Ltd. and the University of Exeter. D. Tran is funded by the Medical Research Future Fund ‐ Cardiovascular Health Mission ‐ Congenital Heart Disease Grant (ARGCHDG000016).

## Disclosures

None.

## Supporting information

Table S1
Figure S1
