# Supplementary material for: Scaling Peak Oxygen Consumption for Body Size and Composition in People With a Fontan Circulation
Source: J Am Heart Assoc. 2022 Dec 14;11(24):e026181. doi: 10.1161/JAHA.122.026181 (PMC9798799; doi:10.1161/JAHA.122.026181)
Supplement: Supplementary file 1 — Table S1 Figure S1 [file JAH3-11-e026181-s001.pdf]

SUPPLEMENTAL MATERIAL

Table S1. Pearson correlation coefficients and 95% CI split by sex.

	Males				Females			
Body size variable	r	n	Lower C.I.	Upper C.I.	r	n	Lower C.I.	Upper C.I.
***Peak VO_2* (mL/min)**								
BM	**0.55**	**42**	**0.29**	**0.73**	**0.34**	**47**	**0.06**	**0.57**
Stature	**0.50**	**42**	**0.23**	**0.70**	**0.40**	**47**	**0.13**	**0.61**
BSA	**0.59**	**42**	**0.35**	**0.76**	**0.42**	**47**	**0.16**	**0.63**
FFM	**0.71**	**38**	**0.50**	**0.84**	**0.52**	**39**	**0.24**	**0.72**
LM	**0.71**	**42**	**0.52**	**0.84**	**0.31**	**47**	**0.03**	**0.55**
ALM	**0.72**	**42**	**0.53**	**0.84**	**0.50**	**47**	**0.24**	**0.69**
Peak VO_2 (Y/X)								
BM	**-0.34**	**42**	**-0.59**	**-0.05**	**-0.40**	**47**	**-0.62**	**-0.13**
Stature	**0.36**	**42**	**0.06**	**0.60**	0.21	47	-0.08	0.47
BSA	0.17	42	-0.14	0.45	0.01	47	-0.27	0.30
FFM	-0.01	38	-0.33	0.31	-0.09	39	-0.39	0.23
LM	0.02	42	-0.28	0.32	-0.26	47	-0.51	0.03
ALM	-0.08	42	-0.38	0.23	-0.17	47	-0.43	0.13
Peak VO_2 (Y/X^b)								
BM	0.02	42	-0.28	0.33	-0.16	47	-0.43	0.13
Stature	0.06	42	-0.25	0.36	-0.12	47	-0.40	0.17
BSA	0.03	42	-0.28	0.33	-0.10	47	-0.38	0.19
FFM	0.04	38	-0.29	0.35	-0.06	39	-0.37	0.26
LM	0.17	42	-0.15	0.45	-0.19	47	-0.45	0.11
ALM	0.07	42	-0.24	0.36	-0.09	47	-0.36	0.21

Figure S1. Scaling peak VO_2 to different body size variables using ratio and allometric scaling.

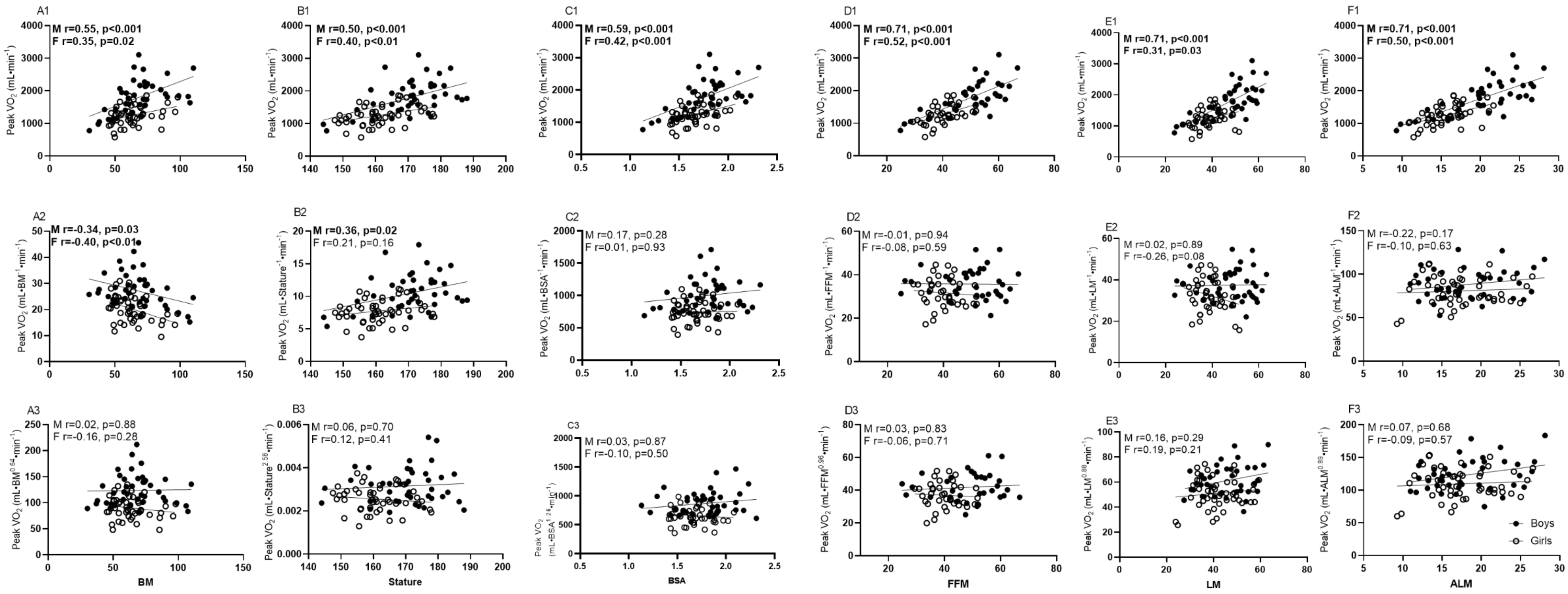

Peak VO_2 (mL/min), absolute oxygen consumption; Peak VO_2 (Y/X), ratio scaled oxygen consumption; Peak VO_2 (Y/X^b), allometrically scaled oxygen consumption; M, Male; F, female; BM, body mass; BSA, body surface area, FFM, fat free mass; LM, lean mass; ALM, appendicular lean mass.
